# Editorial: Advanced biosensors and chemosensors toward mycotoxins analysis for food safety

**DOI:** 10.3389/fnut.2026.2006057

**Published:** 2026-10-02

**Authors:** Mengke Zhang, Xiaodong Guo, Marie-Laure Fauconnier

**Affiliations:** 1 College of Food Science and Light Industry, Nanjing Tech University, Nanjing, China; 2 College of Animal Science and Technology, Yangzhou University, Yangzhou, China; 3 Joint International Research Laboratory of Agriculture & Agri-Product Safety of MOE, Yangzhou University, Yangzhou, China; 4 Chimie Générale et Organique, Gembloux Agro-Bio Tech, Université de Liège, Gembloux, Belgium

**Keywords:** aptamer, aptasensor, detection, food safety, mycotoxins

Food safety remains a global concern for public health, economic stability, and society wellbeing. Mycotoxins, one of the most prevalent and severe classes of contaminants, are toxic secondary metabolites produced by filamentous fungi. Generally, the main mycotoxins are divided into aflatoxins (AF), ochratoxins (OT), fumonisins (F), zearalenone (ZEN), deoxynivalenol (DON), and T-2 toxin, thereby contaminating a wide spectrum of commodities like cereals, maize, peanuts, fruits, oil, milk, and processed foods. Of them, for instance, AFB1, OTA, FB1, ZEN are regulated as group 1, 2B, 3 carcinogens by International Agency for Research on Cancer (IARC), respectively. Considering their serious risks to animal and human health, many countries and organizations including European Union, USA, China etc., have set the maximum residue levels (MRLs) down to ppt levels toward mycotoxins in certain commodities.

Conventional analytical methods, high-performance liquid chromatography coupled with mass spectrometry (HPLC-MS), represent for the gold standard against quantitative detection of mycotoxins. However, high cost, skilled personnel, and time-consuming procedures limit their application scenarios to laboratory. Fortunately, assisted by antibody development, antibody-based immunoassays like enzyme-linked immunosorbent assay (ELISA) and immunosensors have been well established for rapid and on-site food analysis. Nevertheless, several obstacles such as weak reproducibility, false-positive results and complicated protocols remain big challenge. Consequently, there is an urgent demand to exploit novel biosensing and chemosensing platforms that enable fast, sensitive, specific, cost-effective, and point-of-care (POC) testing of mycotoxins and even trace levels.

Very excitingly, our Research Topic entitled “*Advanced biosensors and chemosensors toward mycotoxins analysis for food safety*” is timely and of high significance to food safety, conceiving to overcome the challenges by exploring cutting-edge sensing platform, elucidating the underlying mechanisms and design principles, and revealing their potential for commercialization. Upon the continuous efforts, accordingly, the collection attracts four contributions spanning original research and comprehensive review, advancing our knowledge of mycotoxin detection from molecular recognition to real-world monitoring (Figure 1).

**Figure 1:**
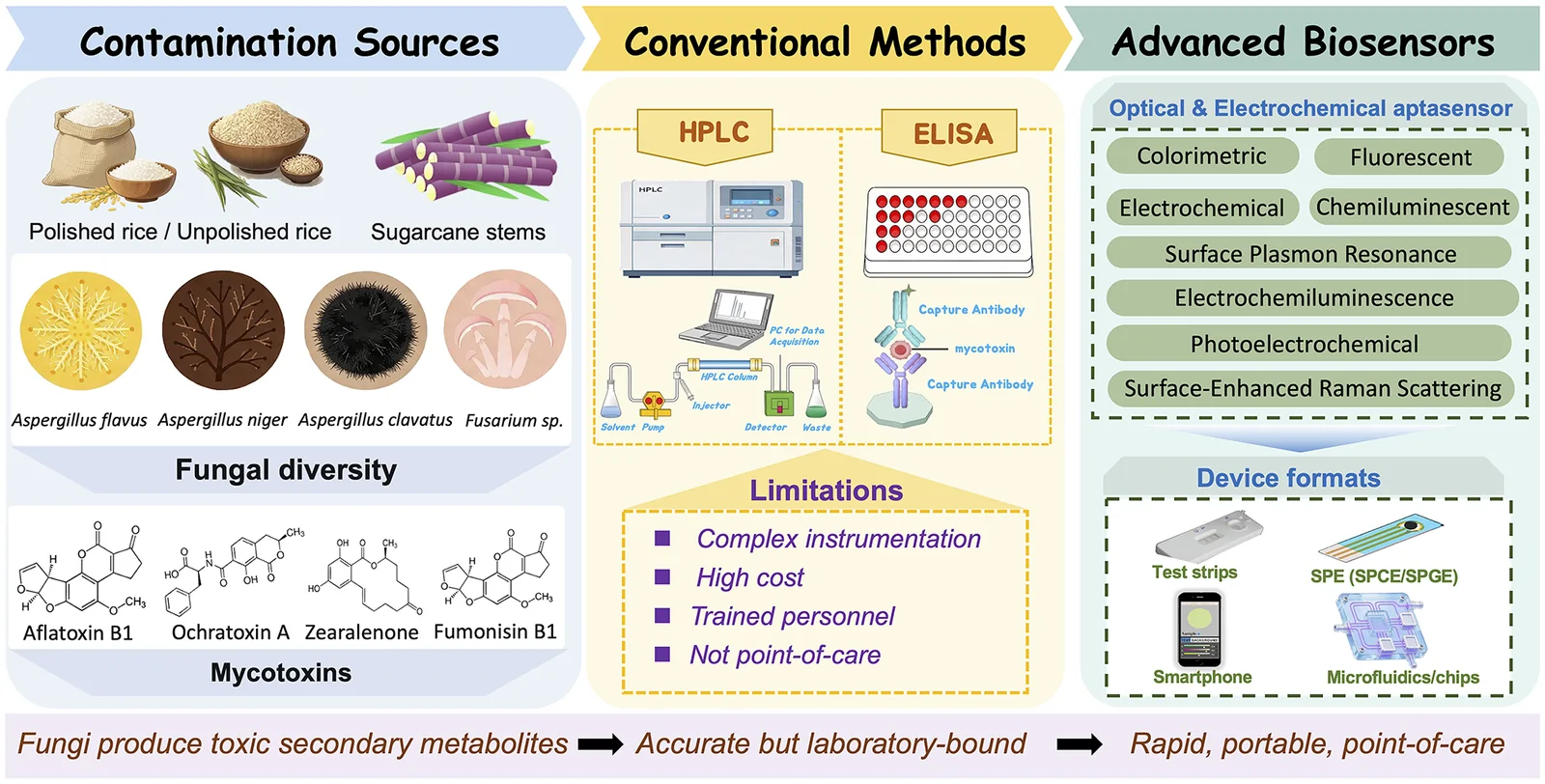
Schematic overview of the Research Topic, illustrating the pathway from fungal contamination of food matrices through mycotoxin production to advanced biosensing and chemosensing strategies, ultimately contributing food safety and human health.

The first contribution introduced by Sánchez-Carrillo et al., presents a colorimetric chemosensor to detect AFB1 based on the aggregation of L-lysine-functionalized gold nanoparticles (AuNPs-Lys) when copper ions were existed. In their design, the synthesized AuNPs-Lys were systematically characterized using UV-Vis, fluorescence, FTIR, nanoparticle tracking analysis, dynamic light scattering, and transmission electron microscopy. The colorimetric aptasensor exhibited a linear response vs. AFB1 concentrations in the range of 6.25–200 ng/mL and a limit of detection (LOD) of 4.18 ng/mL. Compared to antibody-based immunoassays, method validation was completed for recoveries of 91.2%−98.4% in maize samples using the 991.31 AOAC official procedure. This work underscores the potential of nanoparticle-assisted colorimetric sensor as rapid, cost-effective and on-site alternatives for quantification of AFB1 in food.

In order to complement this innovative work, Ma and Guo further provide a comprehensive review on current progress in optical and electrochemical aptasensors for FB1 determination from 2013 to 2025. This article catalogs aptasensor platforms according to the signal transduction strategy including fluorescence, colorimetry, surface-enhanced Raman spectroscopy (SERS), electrochemistry, photoelectrochemistry, electrochemical impedance spectroscopy, and electrochemiluminescence, highlighting the impact of nanomaterial integration, sensor configuration, and signal amplification techniques on analytical performance. Consequently, by overcoming the key problems, we offer a critical roadmap for accelerating aptasensor technology from laboratory to commercialization.

Unlike the development of sensors, two original research articles focus on the fungal diversity and mycotoxin contamination in main food commodities. Firstly, Doungsrikaew et al. report an integrated polyphasic characterization of fungal isolated from sugarcane (Saccharum officinarum) stems in Thailand. Combining morphological research with multilocus phylogenetic analysis and targeted LC-ESI-MS/MS mycotoxin profiling, they identified 64 fungal isolates mainly with Curvularia (57.81%), Fusarium (15.62%), and Nigrospora (9.38%) as the predominant genera. Noteworthy, all Fusarium sacchari isolates produced beauvericin (BEA), and Paecilomyces lecythidis was indicated to synthesize citrinin for the first time. This work highlights the underrecognized toxigenic potential of fungi associated with sugarcane and reveal the requirement of surveillance strategies extending to traditionally monitored aflatoxins.

Furthermore, Waiya et al. investigated fungal diversity and mycotoxin generation in retail polished and unpolished rice resourced from department stores across Bangkok, Thailand. It was demonstrated that 12.6% of 20,000 examined rice grains were contaminated with unpolished rice (13.28%) displaying slightly higher contamination than polished rice (11.92%) by using the blotter method. Morphological and multilocus molecular identification indicated *Aspergillus* as the dominant genus (90% of isolates), together with *Neocosmospora* (formerly Fusarium solani species complex) and *Penicillium/Talaromyces*. Notably, BEA (39.42 and 65.57 μg/kg) and ZEN (0.85 and 1.77 μg/kg) were determined exclusively in unpolished rice samples by LC-MS/MS, while other main mycotoxins like AFB1, OTA, FB1 was below the LODs in certain matrix. This research enhances the status of bran layer as a reservoir for fungal propagules and mycotoxins, thereby improving the application of multiple analytical methods in routine food safety control.

In this Research Topic, we can conclude that the articles focus on the landscape of mycotoxin analysis, spanning from novel nanomaterial-based chemosensors and nucleic acid aptasensors to comprehensive surveys of fungal ecology and mycotoxin occurrence. The systematically integrated protocol plays an important role in mycotoxin control with high efficiency. Specifically, advanced sensing technology should be involved of robust observation data that can recognize contamination hotspots, identify fungal communities, as well as monitor mycotoxins occurrence and levels. Future direction should prioritize the translation of sensing strategies from laboratory to commercialization. In this regard, miniaturized devices including smartphone-based and wearable platforms can integrate to these sensors with versatile and user-friendly format. By bridging principle innovation with real-world application, our topic can contribute to the broader mission of improving food safety and protecting public health.

